# Adaptation of the Kiswahili and Lingala Versions of the LittlEARS^®^ Auditory Questionnaire (LEAQ) in Children with Normal Hearing in the Democratic Republic of Congo (DRC)

**DOI:** 10.3390/audiolres15030054

**Published:** 2025-05-07

**Authors:** Ismael K. Byaruhanga, Frans Coninx, Karolin Schäfer

**Affiliations:** 1 Centre for Education & Community Based Rehabilitation, Arua/Ug, Aru P.O. Box 1364, Democratic Republic of the Congo; 2 Department of Special Education and Rehabilitation, Faculty of Human Sciences, University of Cologne, 50931 Köln, Germany; 3 Institute for Audiopaedagogics, IfAP, 42697 Solingen, Germany; f.coninx@ifap.info; 4 Institute for Special Education Needs (d/Deaf and Hard of Hearing), University of Duisburg-Essen, 45127 Essen, Germany; karolin.schaefer@uni-due.de

**Keywords:** LittlEARS^®^, auditory questionnaire, assessment, adaptation, auditory development

## Abstract

**Background and objective**: Currently, there is no universal newborn hearing (UNHS) screening in the Democratic Republic of Congo (DRC), partly due to financial and organizational reasons. The aim of this study was to adapt the LittlEARS^®^ Auditory Questionnaire (LEAQ) into the Kiswahili and Lingala languages for use as a screening tool for auditory development in children less than two years of age, as objective hearing screening cannot be performed nationwide. **Methods:** For this purpose, norm data were collected from *n* = 723 infants (Lingala) and *n* = 648 infants (Kiswahili) aged between 0 and 24 months in a random selection of four out of seven health zones with pediatric services in the DRC. A non-linear regression model was derived for total scores by age. Pearson correlations were performed to determine whether there were differences between total scores of the questionnaire by region (urban/rural areas). A Mann–Whitney U-test was calculated to determine if there were differences depending on who completed the questionnaire (mother/other caregiver). In addition, a one-way ANOVA was used to determine whether there were differences in the total scores of the questionnaires according to the educational background of the person completing the questionnaire. **Results:** The Pearson correlation coefficient between total scores and age was 0.81 for Kiswahili and 0.77 for Lingala. There were no significant differences in total scores for all conditions tested (total scores by age, language, region, person who completed the questionnaire, educational background). **Conclusions:** The LEAQ can be used effectively in a large region such as the DRC, regardless of the region, respondent, and the educational background.

## 1. Introduction

The LittlEARS^®^ Auditory Questionnaire (LEAQ) was developed to assess the auditory behavior of infants with normal hearing and infants with hearing loss who receive a cochlear implant or hearing aid before 24 months of age [1,2,3]. The LEAQ consists of 35 items to be completed by parents or caregivers to assess the auditory development during the first two years of an infant’s life, including receptive and semantic auditory behavior and early expressive vocal behavior.

The questions are listed by age and can be answered simply with YES or NO. The sum of all “yes” answers provides a score that can be compared to the normative data, i.e., the mean and minimum values for each 1-month age group between 0 and 24 months. The mode of administration of the questionnaire (paper and pencil versus interview) does not affect the results [1].

Several clinical tools have been developed for the assessment of auditory skills in young children, largely as a result of research and clinical developments in the field of cochlear implants (CIs) [4,5,6]. Most of these tools, such as parent questionnaires, have been developed and are available in developed countries.

In a study by Bagatto et al. [7], most of the tools for the assessment of auditory skills in infants were evaluated based on the basis of conceptual clarity, standards, and measurement models, and are available in different languages for people from different cultural backgrounds. Despite the advantages and the statistical integrity of the various hearing-related subjective behavioural tests, there is evidence that results obtained in this manner may not relate well to reports of child behavior in a structured setting. It appears that the applicability of the questionnaires in many research, clinical, and educational settings is limited by the time required to complete the questionnaire and the requirement that the parents be literate. Recent studies show that people from rural areas have less access to health information, mass media, and scientific literature [8,9,10]. Similarly, the results of the Demographic and Health Survey [11,12] show dramatic differences between urban and rural populations, for example, in child mortality rates by maternal education in rural areas (122 deaths per 1000 live births for children of mothers with no education compared with 93 for children of mothers with secondary or higher education). The survey shows that stunting is higher in rural areas than in urban areas (47% vs. 33%) and that the proportion of women with no schooling is more than three times higher than that of men (15% vs. 4%). Therefore, service delivery could be improved to make health information more efficient and effective for rural communities. These limitations may negatively impact the results of hearing assessment of children in developing countries such as the Democratic Republic of Congo (DRC), particularly in rural areas where the literacy rate is low and universal neonatal hearing screening (UNHS) has not yet been implemented [13]. As the implementation of universal hearing screening or other objective diagnostic methods is currently not feasible due to financial and organizational constraints in the DRC, alternative measures such as assessments, observation sheets, or survey tools are necessary to provide an approximation to UNHS. In their study, Schaefer et al. [14] found that the German version of the LEAQ can serve as a secondary hearing screening tool for older children when they reach 12 months of age. Therefore, it seems prudent to be adapt the LEAQ in the national languages, Kiswahili and Lingala.

The LEAQ, which has been adapted to various social and cultural contexts, is available in over 20 languages worldwide, with approximately around 90% being European languages [1,7,9,10,11,12,13,14,15,16,17,18,19]. Furthermore, the LEAQ received an “A” grade for conceptual clarity. Both Coninx et al. [1] and Bagatto et al. [7] concluded that the LEAQ is an effective assessment tool for evaluating auditory development, thanks to its two optional modes of questionnaire administration and other criteria, such as the absence of bias in both the items and the instrument itself. The responses from parents are not influenced by cultural differences or social circumstances. Additionally, the tool demonstrates internal consistency, validity, and sensitivity, along with a high usability in clinical practice [15,16,17].

To date, the only research that exists about the LEAQ in African countries is the adapted version in three Ghanaian languages: Fanti, Akuapem, and Asante [15]. No studies exist about other African languages with many speakers (e.g., the Lingala and Kiswahili languages in the DRC). Therefore, the adaptation of the LEAQ is of utmost importance in African countries where thousands of infants and children are subject to many environmental factors favoring hearing problems and where audiology services are gradually developing; both are particularly true for the DRC.

The aim of this research was to translate and adapt the LEAQ into the African languages Kiswahili and Lingala. The secondary aims of this research are the following: (1) to correlate between the age dependency and norm values of auditory skills for the Lingala and Kiswahili versions of the LEAQ; (2) to differentiate between the auditory behavior observed by caregivers in urban (town/city) and rural areas (village) and also the difference in response patterns between urban and rural areas; (3) to differentiate between the auditory behavior observed by respondents who are mothers (biological) and others (father, siblings, grandmother, and caregivers); (4) to differentiate the total number of “yes” answers (score) in the LEAQ ^®^ given by parents based on their highest educational level, divided into four groups (without education (O), primary education (P), secondary education (S), and university education (U)).

## 2. Materials and Methods

The LittlEARS^®^ Auditory Questionnaire (LEAQ) was originally developed in German [1]. The original German version is considered the basis for any adaptation to other languages. For this study, the English (UK version), as the first translation from the original German version, was used. For adaptation, the recommendations of Hall et al. [18] were followed by using the step-by-step guide for translating hearing-related questionnaires for different languages and cultures. The procedures included identification and selection of the translators (two qualified linguistic teachers from two independent university colleges, Department of Linguistics); translation from the English version into Lingala and Kiswahili and back translation (Person A completed the back translation of B and vice versa without knowing each other or the original version of the questionnaire). The evaluation phase was carried out by the IFAP Institute in Solingen, Germany, to ensure that the translated version of the text was linguistically equivalent to the original English version. To test the administration of the questionnaire, a random selection of four out of seven health zones with pediatric services in North Ituri was performed. Pre-testing and readjustment were performed especially for the Lingala spoken in the northern part of the DRC (in the Haut- and Bas-Uélé Provinces), originally called “Bangala”, to Lingala spoken in the western part. The same procedure was carried out for the Kiswahili spoken in the eastern part of the DRC (Ituri/Bunia Province) to the Kiswahili spoken in the southeast (Goma–Bukavu–Lubumbashi). A total of 60 participants aged between 0 and 24 months were involved, with 15 participants from the four provinces that included Lingala-speaking and Kiswahili-speaking parents. The internal consistency was carried out for both versions, and Cronbach’s alpha coefficient was 0.83 for the Lingala version and 0.90 for the Kiswahili version, which is comparable to the original German version (0.96) [1].

This study was conducted in the Democratic Republic of the Congo (DRC), one of the most linguistically most diverse countries in the world, with over 200 languages spoken in the country. French is the official language followed by four national languages: Kikongo, Lingala, Kiswahili, and Tshiluba. The distribution of speakers of each of the four national languages is mostly clustered in four parts of the country. Lingala has about 14 million speakers and is more widely spoken in the northwest, and Kiswahili, with about 22 million speakers, is more spoken in the east. These two languages (Kiswahili and Lingala) have about 36 million speakers [19].

### 2.1. Participants and Data Collection

The LEAQ was administered to caregivers with normal-hearing children who were mostly biological mothers, fathers, siblings, and grandparents during routine immunization activities and also to caregivers in the pediatric wards. Ear and hearing evaluation was carried out before handing out the questionnaires by an otoscopy checkup to exclude external conditions such as impacted wax, chronic otitis media, or any malformation of the external ear canal. After that, tympanometry was conducted to exclude any other middle ear condition as well as a screening using transient evoked otoacoustic emissions (TE-OAE).

Caregivers who did not attend school were aided by professionals who were requested to only read the question without any additional comment or explanation to prevent parents from being influenced. Ethical approval for the study was obtained from the Provincial Ministry, Health Divisions (Kinshasa, le 16/07/2018; Autorisation de recherche n0: 054/240/DPS/IT/07/2018). The inclusion criterion for the child was being aged between 0 and 24 months. Data were collected from 723 babies and toddlers for the Lingala version and 648 babies and toddlers for the Kiswahili version.

### 2.2. Statistical Analysis

Scale analysis (Pearson’s correlation coefficient) was used to determine the correlation between the total score and the infants’ age and presented as scatterplots; quantitative data are presented as mean (M) and standard deviation (SD). A regression analysis was performed with age as the independent variable and the total score as the dependent variable; this helped to generate a normal curve with standardized values. Pearson correlations were used to obtain the correlation between the responses and the LEAQ scores from categories of respondents. To compare the impact of education level on the LEAQ score, a one-way ANOVA was used. All the data were analyzed using the Statistical Package for the Social Sciences (SPSS) version 20, and statistical significance was set to *p* < 0.05. All this was carried out for both Kiswahili and Lingala.

## 3. Results

**Figure 1:**
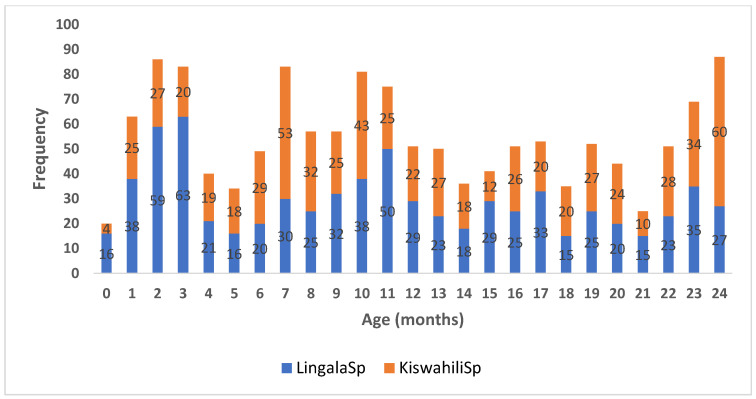
Age distribution in months for Lingala and Kiswahili (Lingala speakers (SP) *n* = 723, Kiswahili speakers (SP) *n* = 648).

A histogram (Figure 1) is used to illustrate the data distribution and the spread of values of the data and to demonstrate outliers [20]. Figure 1 shows that about 85% of the age intervals have an almost equal number of children. This also applies to gender distribution, as 323 girls (49.8%) and 325 boys (50.2%) were involved in the Kiswahili version, and 394 girls (54.5%) and 329 boys (45.5%) were involved in the Lingala version.

**Figure 2:**
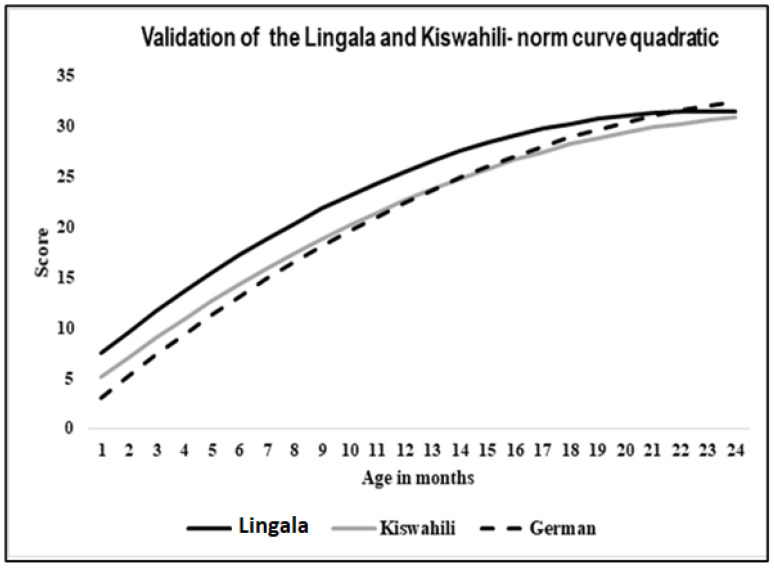
LEAQ validation data for normal hearing children for the Lingala and the Kiswahili languages, compared to the original German version [1].

Figure 2 shows that there is a positive correlation between LEAQ scores and age, i.e., the dependent variable scores increase with age.

The correlation coefficient for the Kiswahili LEAQ version was r = 0.81 (*p* ≤ 0.001); the correlation coefficient for the Lingala LEAQ version was r = 0.77 (*p* ≤ 0.001).

A polynomial regression analysis was conducted, and the regression curve with age as an independent variable and total scores as dependent variables shows that the responses for the Kiswahili version were similar to the German original version, whereas the Lingala version slightly differed from the original German version (see Figure 2 and Table 1). Table 2 shows an overview of the correlation between age and total score for different language versions of the LEAQ.

There was no difference in total scores in both versions between urban (town) and rural (village) areas. No significant correlation was found between the residence of the respondents (urban/rural areas) and the total scores on the Lingala LEAQ version (r = −0.05 (*p* = 0.149)). Similarly, no significant correlation was found between the residence of the respondents (urban/rural areas) and the total scores on the Kiswahili LEAQ version (r = 0.07 (*p* = 0.053)).

A Mann–Whitney U-test was calculated to determine whether there were differences depending on who completed the questionnaire (mother/others) in both language versions of the LEAQ (Kiswahili and Lingala). The total number of biological mothers of Kiswahili infants was *n* = 537; the total number of other caregivers of Kiswahili infants was *n* = 111. There was no statistically significant difference between the two groups (mother/others) and the total scores of the Kiswahili LEAQ version (U = 28646.500, Z = −0.648, *p* = 0.517).

The total number of biological mothers of Lingala infants was *n* = 632; the total number of other caregivers (fathers, siblings, grandmothers) of Lingala infants was *n* = 91. As in the Kiswahili LEAQ version, no significant difference in the total scores was observed in the Lingala LEAQ version, regardless of whether the mother or another person completed the questionnaire (U = 28705.500, Z = −0.027, *p* = 0.978).

Table 3 shows that out of 723 respondents of the Lingala version, 62 (8.6%) had no education level, i.e., they had never been to school; 179 (24.8%) had primary education level; 456 (63%) had secondary education level; 25 (3.5%) had attained university or tertiary level education; and 1 (0.1%) was a missing value.

The respondents’ mean LEAQ scores for primary (M = 21.92) and secondary (M = 21.65) education were slightly lower than the LEAQ scores of respondents without any education level (M = 22.45) and those with a university education level (M = 22.48).

Table 4 shows that out of 648 respondents of the Kiswahili version, 108 (16.7%) had primary education level, 455 (70.2%) had secondary education level, and 85 (13.1%) had attained university or tertiary level education. There was no record for respondents with no education in the Kiswahili group.

A one-way ANOVA was used, and the test did not reveal any significant difference in the LEAQ scores and the educational levels of both the Kiswahili (F (1.4434) = 0.239 *p* = 0.126) and Lingala respondents (F (0.153) = 0.928 *p* = 0.971). Respondents with a university education level (U) (mean = 20.61) had slightly better scores than those with a secondary education level (S) (mean = 22.27) and a primary education level (P) (mean = 23.15).

## 4. Discussion

This study provides the first data-based information on the Lingala and Kiswahili adaptations of the LEAQ as a tool for assessing auditory behavior in babies, infants, and children aged 0–24 months in the DRC.

Results indicate that the adapted Lingala and Kiswahili versions of the LEAQ are appropriate for assessing auditory development in children under 24 months in the DRC. Moreover, the strong correlation found between total scores and age supports the use of the LEAQ in languages spoken in the DRC to monitor early auditory behavior, make informed decisions in time, and obtain an overview of auditory development in children under 24 months.

The high correlation between age and total score in Lingala and Kiswahili is consistent with other studies, first of all with the original German version of the LEAQ [1] and with versions in other languages, such as Fante, Akwapen, and Asante in Ghana, Africa [15], Polish [21], Mandarin [22], English (Canada) [7], Hebrew and Arabic [23], Spanish (South America, Spain) [24,25], Yoruba [26], Finnish [16], Swedish [27], Maltese [28], and Persian [29]. Therefore, it can be assumed that even when the LEAQ is conducted in different multilingual countries, it is still effective and shows similar results. This is likely because the questions are easy to answer and culturally adaptable.

Furthermore, this study shows that the differences in everyday life, which are determined by the residence of the respondents either in a city/town or in a rural area/village, including their lifestyles, have no influence on the mother’s and caregiver’s observations of the auditory development of their children. Also, there was no significant effect of respondents’ educational level on the LEAQ scores. This is in line with studies conducted in Ghana by Offei and Coninx [15]. Moreover, it also confirms that the LEAQ is valid and reliable [1] as it cannot be affected by different backgrounds or educational levels of participants. However, it must be noted that people without education had the support of an interviewer. Although the interviewer was instructed not to intervene in the answering process, it cannot be ruled out that this, nevertheless, had an effect on the answering behavior. These results should, therefore, be interpreted with caution.

With regard to the geographical accessibility, the dispersal of the population, the distances to be traveled to access the health centers (i.e., beyond 5 km or more than an hour’s walk), and natural obstacles, especially rural areas, limit access to health care services. Therefore, the LEAQ is a suitable tool that can contribute to improve community accessibility to hearing health care services. Reaching health centers is a problem for about 48% of mothers in rural areas, in contrast to 25% in urban areas.

The lack of respondents without education level in the Kiswahili-speaking province could be due to the high rate of education compared to the Lingala-speaking provinces, especially in Haut and Bas-Uélé Provinces in the northern DRC. Outliers may originate from the fact that the respondents with no education level had more help from others, and it is possible that they could have expressed some uncertain answers compared to those who were not aided. In addition, the difference in the means observed between the respondents who had primary and secondary level and those with no education and with university level can also be explained by the large disproportion of the number of participants.

This means that the status of the interviewee as a biological mother, or other caregiver, father, siblings, grandmother, maid, etc., has little effect on LEAQ scores for the Lingala and Kiswahili versions. More than 60% of the data were collected from the biological mothers of the children, and about 40% were collected from health centers during routine immunization activities, whereby not only biological mothers are present, but also other family members who are not always present in the children’s everyday lives. This means that the status of the interviewee as a biological mother or other caregiver, father, siblings, grandmother, maid, etc., has little effect on LEAQ scores for the Lingala and Kiswahili versions. This is in line with the study by Offei and Coninx [15] that states that using the LEAQ enables assessment services to be provided to different people in a variety of informal settings. Nevertheless, biological mothers should be involved in the survey to offer them knowledge about the process of the auditory behavior assessment. This will positively impact not only the child in hand but also future children. The more biological mothers are involved and informed about auditory skills for language development, the easier it will be to establish the LEAQ as a welcome alternative in regions where objective hearing screenings are difficult to implement [14].

In a next step, LEAQ versions in Kikongo and Tshiluba should be adapted and validated so that all four national languages are available in the DRC. That may be of great importance to provide guidelines for the application of the questionnaire in primary health care (PHC). Furthermore, as smartphones are widely available not only in cities but also in rural areas, a future evolution of a smartphone-based LEAQ would be useful.

## 5. Conclusions

The Lingala and Kiswahili versions of the LEAQ are useful tools to evaluate the auditory development in children aged 0–24 months. This is particularly important considering that, despite the universal neonatal hearing screening (UNHS) being the gold standard, the procedures for the LEAQ will represent an approximation if UNHS cannot yet be introduced in the DRC for financial and organizational reasons.

## Figures and Tables

**Table 1:** Means, standard deviations and correlations with confidence intervals.

Lingala Version (*n* = 723)				Kiswahili Version (*n* = 648)		
Variables	Score M (Range)	SD	Pearson Correlation Coefficient	Score M (Range)	SD	Pearson Correlation Coefficient
Age	10.92 (0–35)	7.2	0.77 **	12.72 (0–35)	7.25	0.81 **
Scores	21.81	10.53		22.2	10.46	

**. Correlation is significant at the 0.01 level (2-tailed).

**Table 2:** Parameters of the scale analysis: Summary of the correlation between age and total score, global results (Europe, China, Russia, USA, and Africa).

Country (Language)	Sample Size (n)	Corr. Age + Total Score *
Belgium (Flemish)	142	0.93
Bulgaria (Bulgarian)	101	0.82
China (Mandarin)	157	0.84
Finland (Finnish)	364	0.91
France (French)	216	0.83
Germany/Austria (German)	218	0.91
Greece (Greek)	93	0.80
Poland (Polish)	325	0.90
Romania (Romanian)	88	0.80
Russia (Russian)	180	0.93
Serbia (Serbian)	183	0.86
Slovakia (Slovak)	592	0.92
Slovenia (Slovenian)	366	0.92
Switzerland (German)	92	0.92
Malta (Maltese)	398	0.90
USA (English)	144	0.85
USA (Spanish)	48	0.93
Overall	3309	0.89
DRC/Africa (Lingala)	723	0.77
DRC/Africa (Kiswahili)	648	0.81

* Pearson’s correlation coefficient ranges from 0 to 1, where a score of 0.7 or higher shows a high correlation.

**Table 3:** Mean and total number of respondents by educational level for the Lingala version (*n* = 723).

Dependent Variables	N	Mean
Others (with no education) (O)	62	22.45
2. Primary (P)	179	21.92
3. Secondary (S)	456	21.65
4. University (U) (highest education level)	25	22.48

**Table 4:** Mean and total number of respondents by educational level for the Kiswahili version (*n* = 648).

Dependent Variables	N	Mean
1. Secondary (S)	455	22.27
2. Primary (P)	108	23.15
3. University (U)	85	20.61

## Data Availability

The data presented in this study are available on request from the corresponding author.
